# Field-scale robotic phenotyping of three-dimensional wheat canopy architectural traits

**DOI:** 10.3389/fpls.2026.1856006

**Published:** 2026-07-01

**Authors:** Yoon J. Cho, Velu Govindan, Rob Lloyd, Simon Pearson, Oorbessy Gaju, Ravi Valluru

**Affiliations:** 1 Lincoln Institute for Agri-Food Technology (LIAT), University of Lincoln, Lincoln, United Kingdom; 2 International Maize and Wheat Improvement Center (CIMMYT), Texcoco, Estado de México, Mexico; 3 AgriFoRwArds CDT, University of Lincoln, Lincoln, United Kingdom

**Keywords:** 3D models, canopy-based HTP, plant architecture, robotic phenotyping, wheat

## Abstract

Assessing tools for rapid evaluation of new cultivars across large fields is essential for improving crop yields and ensuring future food security. Current phenotyping approaches remain labor-intensive and imprecise at the field scale, particularly for traits determining light interception and the light extinction coefficient (*K*). While robotic phenotyping and three-dimensional (3D) models have gained interest in estimating light interception in plant canopies, primarily at the single-plant scale or using single-plant-derived virtual canopies, applications at the field-scale canopy level remain limited. In this study, a semi-automated robotic phenotyping platform, PhenoLinc, was deployed to obtain canopy-level multispectral 3D data across 200 diverse wheat genotypes grown under field conditions over two years. 3D canopy architecture revealed substantial genotype-specific variation in inclination angle and *K*, which influenced radiation use efficiency (RUE), contrasting with the constant *K* commonly assumed in conventional approaches. This architectural variation was classified as two distinct architectural phenotypes, erectophiles (median angle 65°) and planophiles (59°) at pre-anthesis stages, which converged toward being homogenous by the anthesis stage. Erectophile phenotypes exhibited higher RUE (33.1%) than planophile phenotypes, leading to a higher yield. In yield prediction analyses, 3D-derived architectural traits provided comparable predictive performance to conventional measurements, however, architectural phenotype information reduced prediction error. Together, these findings highlight the value of field-scale robotic canopy phenotyping for characterizing genotype-specific canopy architectural traits and their relationship with yield.

## 1 Introduction

Global food insecurity poses a significant challenge as the world’s population is projected to surpass 10.4 billion by the 2080s (United Nations, 2024). Bread wheat (*Triticum aestivum* L.) is a globally important staple crop with an annual production of approximately ∼808 million tons in 2022 (FAOSTAT, 2023). However, the expected yields are insufficient to meet global wheat consumption (Curtis et al., 2002; Dixon et al., 2009), necessitating a 70% increase in production by 2050 despite stagnating yield caused by climate change and lack of additional arable land (World Health Organization, 2022). Under potential growing conditions, yield can be increased by incident photosynthetically active radiation (PAR), the fraction of intercepted PAR (FiPAR), radiation use efficiency (RUE), and the ratio of grain yield to total above-ground biomass (Harvest Index, HI) (Equation 1) (Reynolds et al., 2017). Since HI has reached a plateau of 0.6, new cultivars with enhanced expression of yield-associated traits, particularly RUE, have been targeted to improve grain yield in favorable environments (Parry et al., 2011).

(1) Yield=PARi x FiPAR x RUE x HI

RUE is one of the crucial traits determining crop yield. It has traditionally been estimated as a coefficient that relates biomass accumulation to cumulative daily intercepted PAR between crop growth stages (Monteith, 1977). RUE depends on factors such as growth stages, light interception, nutrient status, plant architecture, and plant genetics (Sinclair and Muchow, 1999; Yuan et al., 2019). It has been reported that RUE could account for approximately 40% of the variation in yield (Ainsworth and Long, 2021; Long et al., 2006; Molero et al., 2019). Notably, even small increases in RUE and canopy photosynthesis can significantly affect grain yield, particularly when further increases in HI are unlikely (Park and Runkle, 2017). RUE exhibited a gradual annual increase from 2003 to 2016, with an average annual improvement rate of 0.91% (Gerard et al., 2024). However, RUE in current wheat cultivars remains well below its theoretical potential, reporting that it can be substantially improved up to 3.1 g/MJ when considering above-ground biomass (AGB), and up to 4.1 g/MJ including root biomass (Hayman et al., 2024; Loomis and Amthor, 1999; Reynolds et al., 2012). Current methods are also more prone to errors due to the subjective manual measurements, highlighting the need for accurate and efficient methods for estimating RUE and related traits to accelerate the exploration of the genetic diversity of RUE across crop germplasms.

Plant architectural parameters (e.g. leaf inclination angle, leaf area, and plant density) play a critical role in optimizing the light distribution within the canopy, thereby enhancing RUE (Song et al., 2016). Those parameters are crucial for determining grain number and final yield (Richards et al., 2019). In particular, modern wheat cultivars have demonstrated higher RUE compared to older genotypes, which may be due to the optimized canopy architecture (Yunusa et al., 1993). FiPAR, closely linked to plant architecture and density, can be described by Beer-Lambert’s law as a nonlinear relationship between the canopy light extinction coefficient (*K*) and leaf area index (LAI) (Equation 2). *K* is one of the main factors representing the vertical light distribution captured by the canopy, indicating the intensity of light attenuation. Under optimal nutritional and soil-moisture conditions, *K* varies among genotypes and plant densities, depending on plant architecture, such as leaf inclination angle (Maddonni et al., 2001). Erect leaves tend to produce lower *K* values, allowing more light to penetrate into lower canopy layers and reducing light saturation in the upper canopy. This more homogeneous vertical light distribution can enhance RUE. For example, modern genotypes of rice and wheat exhibit erect leaves at the top of the canopy and more floppy leaves in the lower layers to optimize light capture (Mantilla-Perez and Salas Fernandez, 2017; Ort et al., 2015). Consistently, erectophile phenotypes exhibit a 24-28% higher RUE compared to less erectophile phenotypes at the same latitude (Long et al., 2006; Richards et al., 2019). Accordingly, determining plant architecture traits such as leaf distribution and leaf angles could enhance the precision of *K* and FiPAR estimations. However, precise estimation of *K* remains challenging due to the inherent complexity of plant architecture, particularly under field conditions. Traditionally, leaf angles have been measured manually using a protractor and a reference point, which is labor-intensive and impractical under field conditions. In addition, despite variations among wheat genotypes, *K* has been regarded as a constant approximation to represent a spherical leaf distribution, simplifying its use in crop modelling (Baldocchi et al., 1985; Brown et al., 2018; Liu et al., 2021). Using a constant *K* value in heterogeneous canopies could lead to a 17-24% difference in FiPAR estimations, as demonstrated by (Sarlikioti et al., 2011). Therefore, an efficient and accurate approach to quantify plant architectural traits and *K* is essential for the precise estimation of FiPAR and RUE under field conditions.

(2) $$\text{FiPAR}=1–{\text{e}}^{-K\cdot \text{LAI}}$$

Plant phenotyping has traditionally relied on manual measurements and handheld proximal sensors, such as light ceptometers, chlorophyll meters, fluorometers, NDVI sensors, porometers and thermal sensors, to assess canopy structure and physiological performance. Although useful for targeted trait measurement, these methods are often labour-intensive and time-consuming, limiting the number of genotypes and measurement frequency that can be assessed in large breeding populations. Recent advances in high-throughput phenotyping (HTP) have addressed these limitations by using automated sensor-based platforms, including ground vehicles, gantry systems, UAVs and robotic platforms, to collect morphological, physiological and spectral information non-destructively across crop growth stages. These approaches provide efficient tools for identifying plant architectural traits and supporting the selection of breeding lines for yield improvement (Burgess et al., 2021; Chang et al., 2022). Ground-based robotic platforms further enable precise and repeatable proximal measurements under field conditions, supporting fine-scale assessment of canopy structure. However, UAV-based approaches can be constrained by payload capacity, flight regulations, image resolution and limited access to within-canopy structural information, while ground-based platforms can be limited by slower field throughput, battery life and operational complexity. These advantages and limitations highlight the need for field-based robotic phenotyping approaches that can capture fine-scale 3D canopy architecture and link structural traits with physiological processes such as light interception and radiation use efficiency (Atefi et al., 2021; Chawade et al., 2019; Xu and Li, 2022).

3D imaging techniques have been proposed to estimate the leaf inclination angle distribution (LAD) using a 2D stereo imaging system based on a single plant. However, only a limited number of studies have focused on the plot-scale phenotyping in cereal crops Following De Wit (1965), LAD was estimated based on mathematical considerations of the Beta distribution of leaf angle and then classified into six theoretical distributions. For a variety of vegetation canopies, a two-parameter Beta distribution has been considered as the most appropriate approach (Goel and Strebel, 1984; Wang et al., 2007). This distribution has been applied to obtain explicit canopy LAD and the *K* using digital photographic and 3D structural models (Bailey and Mahaffee, 2017; Jin et al., 2016; Pisek et al., 2011; Raabe et al., 2015; Ryu et al., 2010). Therefore, alternative digital approaches are essential for reducing labor-intensive manual measurements while enabling time-effective assessment of canopy architectural traits. In particular, 3D canopy models can detect subtle trait variation and support the rapid exploitation of germplasm for crop improvement (Burgess et al., 2017; Dandrifosse et al., 2020; Foo et al., 2020).

This study aims to leverage field-scale 3D canopy models to explore spatial and temporal variation in canopy architectural traits, such as light interception, *K*, inclination angles, and RUE, across 200 diverse wheat genotypes under field conditions. While these traits have conventionally been measured manually or assumed as constants, HTP tools and 3D imaging enable rapid, precise characterization across large populations under field conditions. This approach addresses key limitations of manual methods by reducing labor intensity and detecting subtle architectural variation associated with improved light interception and RUE.

## 2 Materials and methods

### 2.1 Plant materials and field experiments

Two field experiments were conducted at the Riseholme campus, University of Lincoln, Riseholme, UK (53°15’47.6” N, 0°32’04.6 “W) (**Supplementary Figure 1**). Two diverse sets of 98 spring wheat genotypes (98 genotypes in 2022 and 98 genotypes in 2023) were chosen for this study. The 2022 and 2023 datasets were analyzed separately. These genotypes represent advanced breeding lines obtained from the International Maize and Wheat Improvement Center (CIMMYT). Two genotypes, Cochise and Kilburn, obtained from KWS UK LTD, were used as local checks (n = 200 plots per year). All spring wheat genotypes were cultivated under open field conditions during the spring season of March-August 2022 and April-July 2023. The plots were arranged in a randomized lattice design, with two replicates in 3 m-long, 1.5 m-wide plots, an inter-row spacing of 0.15 m, and a plant density of 250 plants per m^2^. The soil at this site was classified as sandy loam and was slightly alkaline as surveyed in 2023.The trial was well managed in accordance with standard agronomic practices and fertilizer requirements and was maintained under weed-free conditions. 3D canopy data were collected at the heading (GS55) and anthesis (GS65) stages in 2022 and at the booting (GS43) and anthesis (GS65) stages in 2023, following Zadok’s growth scale (Zadoks et al., 1974), when 50% of the plants in the plot reached the respective growth stages.

**Figure 1:**
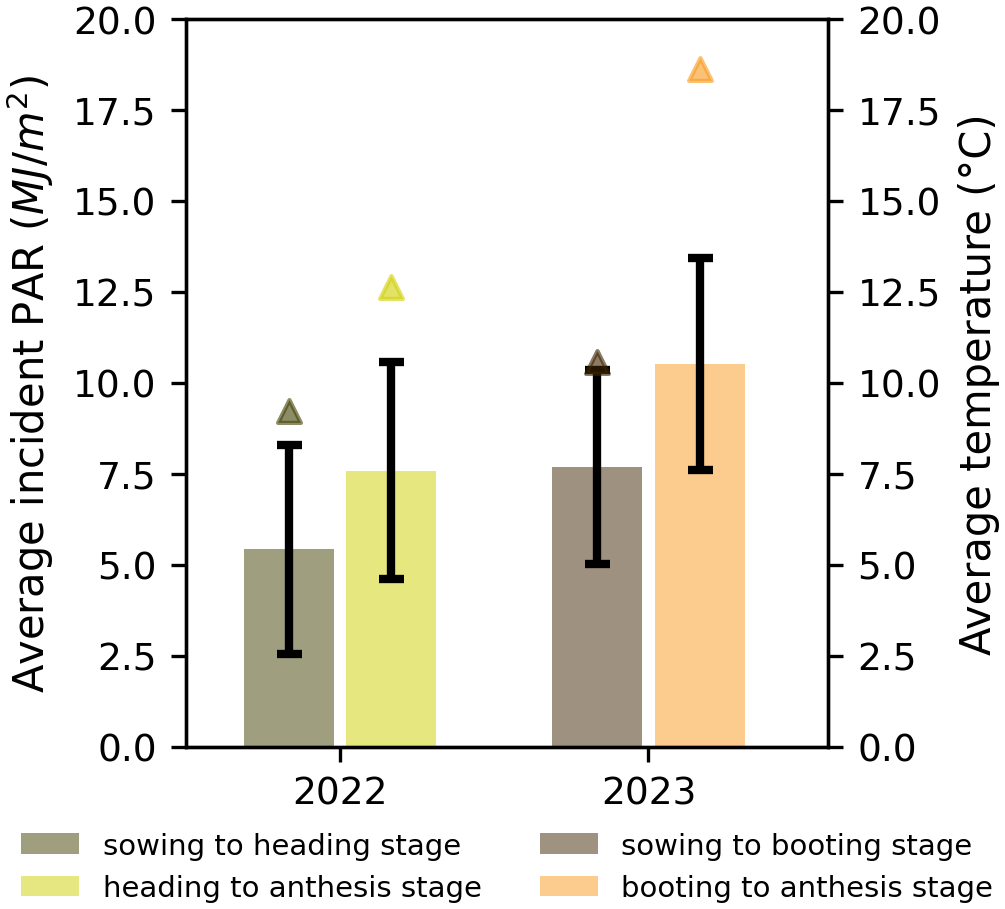
Average incident PAR (bar plot) and temperature (▴) for two physiological growth periods (sowing to heading and heading to anthesis stages in 2022; sowing to booting and booting to anthesis stages in 2023). Error bars represent standard error.

The mean daily solar radiation during the experiment period was collected from a weather station near the field trial site on the Riseholme campus, approximately 580 m away in 2022 and 500 m away in 2023. (**Supplementary Figure 1**). The average incident PAR and temperature across phenological stages over two years are shown in **Figure 1**, which shows a substantial variation between the two years. On average, incident PAR was 28.5% lower, and temperature was 25.06% lower in 2022 than in 2023. Further, the period from sowing to heading (in 2022) and sowing to booting stage (in 2023) had a lower average incident PAR (28.46%, *p* < 0.05 in 2022 and 26.9%, *p* < 0.002 in 2023) and temperatures (26.96% in 2022 and 43.21% in 2023, *p* < 0.001) compared to the period from heading or booting to anthesis stages in 2022 and 2023, respectively. Both incident PAR and temperatures were lower during the early growth stages compared to the anthesis stages.

### 2.2 Robotic phenotyping platform and 3D canopy data collection

**Figure 2:**
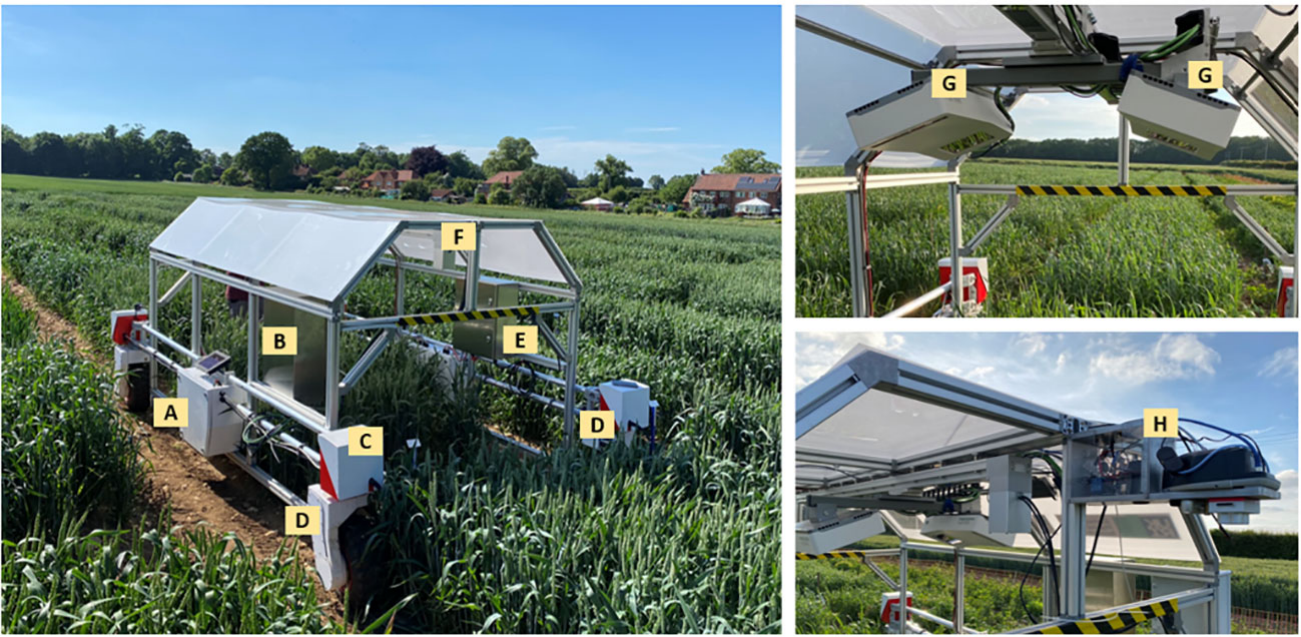
Configuration of the phenotyping platform (PhenoLinc). **(A)** Computer desktop, **(B)** Power system, **(C, D)**. Wheel system, **(E)** Phenospex controller, **(F)** Phenospex rail, **(G)** Dual Phenospex laser scanners, **(H)** Hyperspectral, Multispectral, and IR camera suite.

In both field experiments, a mobile, semi-automated robotic phenotyping platform, PhenoLinc, was deployed to collect 3D canopy data (Cho et al., 2024; Pearson et al., 2022). This phenotyping platform **Figure 2**) is based on the Saga Thorvald II (Grimstad and From, 2017), featuring a reconfigurable modular design and a four-wheel drive system, providing good traction in rough terrains. This is a semi-automated robotic phenotyping platform with a 3 m long and a track width of 1.5 m, allowing passing over fully-grown cereal crops. This fully electric platform runs on 48V lithium-ion batteries, which last up to 8 hours under field conditions. The robot’s modules can be further tailored to suit specific tasks and spatial arrangements, including polytunnels, glasshouses, or field conditions (Pearson et al., 2022). Computation is executed using the open-source Robot Operating System (ROS) on a mini-PC (Intel NUC 8i5BEK mini-PC). This mini-PC connects all sensors to a central network, enabling data capture and automatic storage. The phenotyping platform was operated parallel to the crop rows to avoid crop damage.

Dual 3D multispectral laser scanners (PlantEye F500, Phenospex, Heerlen, the Netherlands) are mounted on a rail system within the platform to capture 3D point-cloud data (laser reflectance at 940 nm). They have a spectral sensitivity of 380–900 nm, covering four spectral bands: red (R, 624-634nm), green (G, 530–540 nm), blue (B, 460-485nm), and near-infrared (NIR, 820–850 nm). These scanners were operated using Phenospex-owned proprietary software, HortControl, to capture and save 3D point cloud data (Kempthorne et al., 2014; Paul et al., 2022). These PlantEye laser scanners have been widely used in plant research to derive numerous plant traits in both controlled and field conditions (Hill et al., 2024; Paladini et al., 2025; Zieschank and Junker, 2023).

### 2.3 3D canopy data processing and derivation of canopy traits

After obtaining the plot-scale 3D data using dual laser scanners, the 3D data were pre-processed by HortControl (v3.8, Phenospex, Heerlen, Netherlands) (Phenospex) to generate the canopy meshes for each plot. Since a dual-scanner system was used for 3D data collection, which provides two 3D meshes (one per scanner), both meshes were merged in CloudCompare (v2.13.alpha, GPL software) to obtain a high-resolution single 3D mesh, which was then used to derive several canopy traits.

The high-resolution canopy mesh (from 3D point cloud data) was further segmented using NDVI (Normalized Difference Vegetation Index) and NIR values to remove noise (e.g., soil-based points - (Mzid et al., 2021; Žížala et al., 2019)) from plant points. A region of interest (ROI) was then defined at the center of each plot, 0.5 m wide and 0.5 m long, and extracted from the entire 3D point cloud without cropping along the z-axis, leaving the full canopy height for further processing. Preprocessing and analysis of the 3D data were performed in Python 3.8 (Python Software Foundation, USA) using custom scripts. The distributions of the number of facets at each developmental stage in 2022 and 2023 are illustrated in **Supplementary Figure 2**.

Using the 3D mini-mesh (0.5m x 0.5m) of each plot, LAD (χ) was estimated by calculating canopy inclination angles (in degrees) for each facet (triangle) (Equation 3), defined as the angle between converted normal vectors ( $\vec{N}$) and a zenith vector ( $\vec{z}$: 0,0,5).

(3) $$\chi =\frac{1}{\theta_{L}}\sum_{t=0}^{\theta_{L}}f(t)$$

Where f(t) is the Beta distribution probability density at t, and $t=\frac{2\theta_{L}}{\pi },\;0<\theta_{L}\leq 90{}^{\circ}$.

Each triangle in the canopy could have its normal vector pointing either upward toward the sky or downward toward the ground. Nevertheless, empirical field measurements indicate that nearly all normal vectors associated with the canopy predominantly point towards the sky (Zheng and Moskal, 2012). Consequently, to ensure all vectors aligned with the sky, the vectors originally pointing in the negative direction towards the ground were assigned reversed values. In Equation 4, normal vectors having negative z-axis components were multiplied by -1 to point them upwards (Zheng and Moskal, 2012).

(4) $${\vec{N}}_{z}=\{\begin{array}{ccc} \;\vec{N_{z}} & if\;\vec{N_{z}}\;\geq 0, & \vec{N_{z}}\;\;\;\;\;\\ -\vec{N_{z}} & if\;\vec{N_{z}}\;<0, & \vec{N_{z}}\times -1 \end{array}$$

Only angles calculated from the top two-thirds of each 3D canopy model were included. This filtering reduces noise from lower canopy layers where occlusion and shadowing effects are greater. These angles were multiplied by 180/π, following (Zheng and Moskal, 2012). The canopy inclination angle ( $0<\theta_{L}\leq 90$) was then computed as Equation 5:

(5) $$\theta_{L}={\text{cos}}^{-1}(\frac{\vec{z}\cdot {\vec{N}}_{\left(x,y,z\right)}}{∥\vec{z}∥∥{\vec{N}}_{\left(x,y,z\right)}∥})\times (\frac{180}{\pi })$$

The 3D canopy data processing pipeline for calculating canopy inclination angles was validated against manual leaf angle measurements using a protractor on a subset of randomly chosen genotypes to ensure the accuracy of the pipeline before field trials (**Supplementary Figure 3**). Manual leaf inclination angles were measured from the vertical using a protractor on the printed 2D images derived from the 3D model (Mantilla-Perez and Salas Fernandez, 2017). Three leaves per plant were scored for leaf angles. At the pot level, the 3D-derived inclination angles showed a small mean bias of 0.72° relative to manual measurements, indicating limited systematic over- or underestimation by the 3D pipeline. The RMSE was 5.52°, which was slightly higher but broadly comparable to the root-mean-square error (RMSE) values of the leaf angle reported for 3D digitizing in maize phenotyping (4.89-4.97°; (Wang et al., 2018)). The canopy inclination angles derived from the 3D models were fitted with the two-parameter based beta distribution function (Equations 1–3), described as the most accurate distribution of the probability density of canopy inclination angle ( $\theta_{L}$) in plant canopies (Wang et al., 2007).

Gaussian mixture model (GMM) is an unsupervised probabilistic clustering technique that models the data distribution as a combination of multiple Gaussian components. In this study, GMM was applied to the full 3D-derived canopy inclination angle probability density distribution from 0° to 90°, obtained from the beta distribution fitted for each genotype. The number of components was set to two *a priori* to compare two biologically meaningful wheat canopy architectures: relatively erectophile and planophile phenotypes (Richards et al., 2019). Genotypes with higher probability density at steeper canopy inclination angles were classified as erectophile phenotypes, whereas those with greater probability density at lower inclination angles were classified as planophile phenotypes, representing more horizontal canopy structures (Huang et al., 2006). Clustering was performed separately for each year using pre-anthesis data, and the resulting PA phenotype groups were subsequently used to evaluate differences in FiPAR, RUE, and yield-related traits at pre-anthesis and anthesis.

Both canopy LAD and solar zenith angle can strongly influence *K* and FiPAR. Hence, before calculating 3D-derived FiPAR (FiPAR_3D_), *K* based on 3D canopy models (*K*_3D_) was computed using LAD derived from the 3D canopy models. The solar zenith angle was calculated using the Helios program (Bailey, 2019), which applies standard astronomical relationships as described by (Iqbal, 1983). Its average value at the time of data collection was used to derive *K* and FiPAR for each genotype. These values were then used to compare effects on the canopy inclination angle distribution between 3D and manual measurements. The genotype-specific beta distribution calculated from the 3D canopy data was considered as the canopy inclination angle distribution in this study, and *K*_3D_ was calculated as Equation 6:

(6) $$K_{3D}=\frac{\sqrt{\chi^{2}+{\text{tan}}^{2}\theta }}{\chi +1.744{\left(\chi +1.182\right)}^{-0.733}}$$

Where χ is the canopy inclination angle distribution (beta distribution) calculated from 3D canopy models for each genotype separately. θ is the solar zenith angle using the Helios (Bailey, 2019).

*K*_3D_ was then compared with the conventional method (*K*_LP_), which was calculated using a fixed χ of 0.96 for wheat (Campbell, 1986; Pokovai and Fodor, 2019). Using *K*_3D_ and *K*_LP_, the FiPAR_3D_ and LP80-based FiPAR (FiPAR_LP_) were calculated, respectively, as Equation 7 and 8:

(7) $${\text{FiPAR}}_{3\text{D}}=1-e^{-K_{3D}\cdot \text{LAI}}$$

(8) $${\text{FiPAR}}_{\text{LP}}=1-e^{-K_{LP}\cdot \text{LAI}}$$

Where FiPAR_3D_ denotes FiPAR derived from the 3d plant model, and FiPAR_LP_ represents FiPAR derived from the conventional LP80-based method.

These FiPAR values were further used to estimate the cumulative intercepted PAR for two growth periods (sowing-to-heading and sowing-to-anthesis in 2022, and sowing-to-booting and sowing-to-anthesis in 2023). The daily mean solar radiation was used to calculate the accumulated intercepted PAR by multiplying the cumulative incident solar radiation by 0.47 (Monteith, 1972) for the respective growth periods in 2022 and 2023. These cumulative intercepted PAR derived from the 3D plant model and LP80 measurements (iPAR_3D_ and iPAR_LP_, respectively) were subsequently used to estimate 3D-derived RUE (RUE_3D_) and LP80-based (RUE_LP_), following Equation 9 and 10:

(9) $$RUE_{3D}=\frac{AGB_{d}-AGB_{d-1}}{iPAR_{3D(d)}-iPAR_{3D(d-1)}}$$

(10) $$RUE_{LP}=\frac{AGB_{d}-AGB_{d-1}}{iPAR_{LP(d)}-iPAR_{LP(d-1)}}$$

Where RUE_3D_ and RUE_LP_ denote RUE derived from the 3D plant model and the conventional LP80-based method, respectively. $AGB_{d}$ and $AGB_{d-1}$are dry above-ground biomass at the determined days *d* and *d-1*, respectively, and $iPAR_{d}$ and *iPAR*_d-1_ denote the cumulative intercepted PAR on the corresponding days.

### 2.4 Ground truth measurements

All measurements were taken around solar noon under cloudless sky and low wind velocity conditions. Measurements were performed between 10:00 and 15:00 to collect stable and accurate measurements (Evans and Santiago, 2014). LAI was collected using an ACCUPAR LP-80 ceptometer (Meter Group, Inc., Pullman, WA, USA) with the PAR wavebands (400–700 nm) by holding it above and below the canopy at a diagonal position across the plant rows for all plots.

AGB was harvested by cutting an entire plant at the ground level within a quadrat (0.5 m × 0.5 m). Subsequently, the harvested biomass was dried in an oven set at 70 °Cfor 48 hours to obtain the dry biomass weight. The distribution of AGB for 2022 and 2023 is represented in the **Supplementary Figure 4**. HI and grain yield were harvested at physiological maturity (GS87) and calculated per m^2^ for each plot. These spikes were then oven-dried at a lower temperature of 50 °Cfor 48 hours before threshing. The resultant grain weight was also used to compute the HI, using the following Equation 11:

(11) $$\text{HI}=\text{Grain weight}(\text{g}/{\text{m}}^{2})/\text{Total biomass}(\text{g}/{\text{m}}^{2})$$

### 2.5 Statistical analysis

All statistical analyses were performed in Python 3.8 (Python Software Foundation, USA) and R Studio 4.2 (RStudio, Inc., Boston, USA). Two PA groups (erectophiles vs. planophiles) were evaluated using Welch’s t-test, implemented through the ttest_ind function in the SciPy library (v 1.9.1), with unequal variances assumed. This approach was selected because the architectural groups were classified using GMM and had unequal sample sizes.

The best linear unbiased predictors (BLUPs) were estimated for all phenotypic traits and then used to fit a random forest (RF) regression model to predict grain yield. 80% of the data was used as a training set, and 20% as a test set to build and validate the RF models. RF was selected because grain yield is influenced by nonlinear and interacting effects among FiPAR, RUE, HI, and canopy architectural traits. RF offers simpler interpretation and reduces overfitting risk through ensemble averaging and provides variable importance estimates (Parmley et al., 2019), allowing the contribution of 3D-derived traits to be assessed within a consistent modelling framework. Four yield prediction models were developed using Equation 12–15:

(12) $$\text{Model }1(\text{M}1):\text{ Yield}∼{\text{FiPAR}}_{3\text{D}}+{\text{RUE}}_{3\text{D}}+\text{HI}$$

(13) $$Model 2(M2):\text{ Yield}∼{\text{FiPAR}}_{\text{LP}}+{\text{RUE}}_{\text{LP}}+\text{HI}$$

(14) $$Model 3(M3):\text{ Yield}∼{\text{FiPAR}}_{3\text{D}}+{\text{RUE}}_{3\text{D}}+\text{HI}+{\text{PA}}_{3\text{D}}$$

(15) $$Model 4(M4):\text{ Yield}∼{\text{FiPAR}}_{\text{LP}}+{\text{RUE}}_{\text{LP}}+\text{HI}+{\text{PA}}_{3\text{D}}$$

These four models, each comprising 30 repetitions, were evaluated to assess their performance. To optimize the model parameters, hyperparameter tuning was conducted using Grid-SearchCV with 5-fold cross-validation based on Model 2 and the resulting optimal configuration was subsequently applied to all four models. Each model’s performance was evaluated using RMSE and the coefficient of determination (R^2^). The highest R^2^ and lowest RMSE values were averaged across the eight sets for each model. R^2^ and RMSE were calculated as were calculated following Equation 16 and17:

(16) $$R^{2}=1-\frac{\sum {\left({\hat{y}}_{i}y_{i}\right)}^{2}}{\sum {\left({\hat{y}}_{i}{\bar{y}}_{i}\right)}^{2}}$$

(17) $$RMSE=\sqrt{\frac{1}{n}\sum_{i=1}^{n}{\left({\hat{y}}_{i}y_{i}\right)}^{2}}$$

Where ${\hat{y}}_{i}$ and ${\bar{y}}_{i}$ are the predicted and mean value, respectively, $y_{i}$is the observed value, and *n* is the total number of observations.

Broad sense heritability (H^2^) was calculated according to Equation 18 following Cullis method (Cullis et al., 2006) with the package ‘inti’ (Lozano-Isla, 2021) in R studio 4.2 (RStudio, Inc. Boston, USA) as:

(18) $$H_{Cullis}^{2}=1-\frac{{\bar{V}}_{\Delta }^{BLUP}}{2\cdot \delta_{G}^{2}}$$

Where ${\bar{V}}_{\Delta }^{BLUP}$ is the mean variance of genotypic BLUPs and $\delta_{G}^{2}$ is genetic variance.

## 3 Results

### 3.1 Phenotypic variation in *K_3D_* leads to trait estimates comparable to LP-based measurement

**Figure 3:**
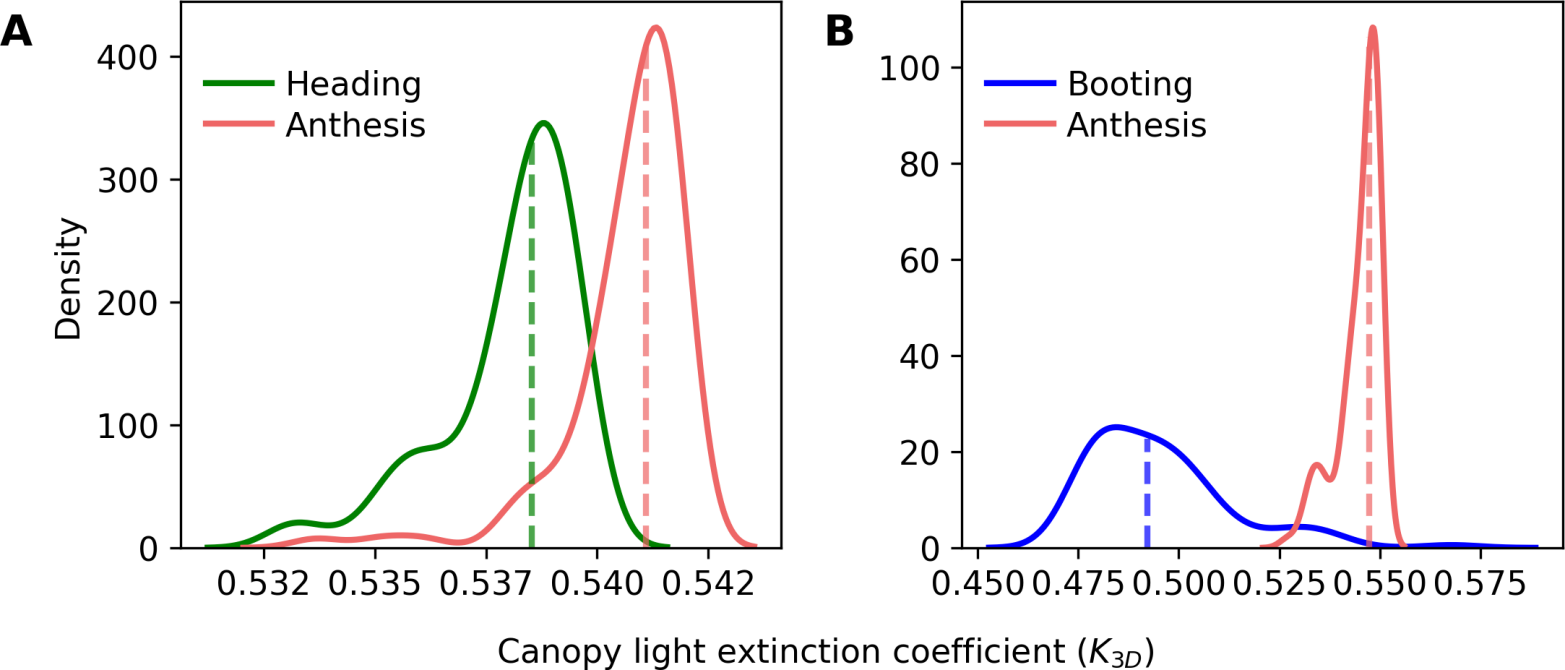
Canopy light extinction coefficient at two growth stages [heading and anthesis in 2022 **(A)** and booting and anthesis in 2023 **(B)**]. The vertical dotted line represents the median value of all genotypes.

*K*_3D_ showed a larger variation in all growth stages compared to the constant value of the *K*_LP_ (**Figure 3**). Across growth stages, *K*_3D_ was higher at anthesis stage than at pre-anthesis stage by 0.47% and 11.23% in 2022 and 2023, respectively.

**Figure 4:**
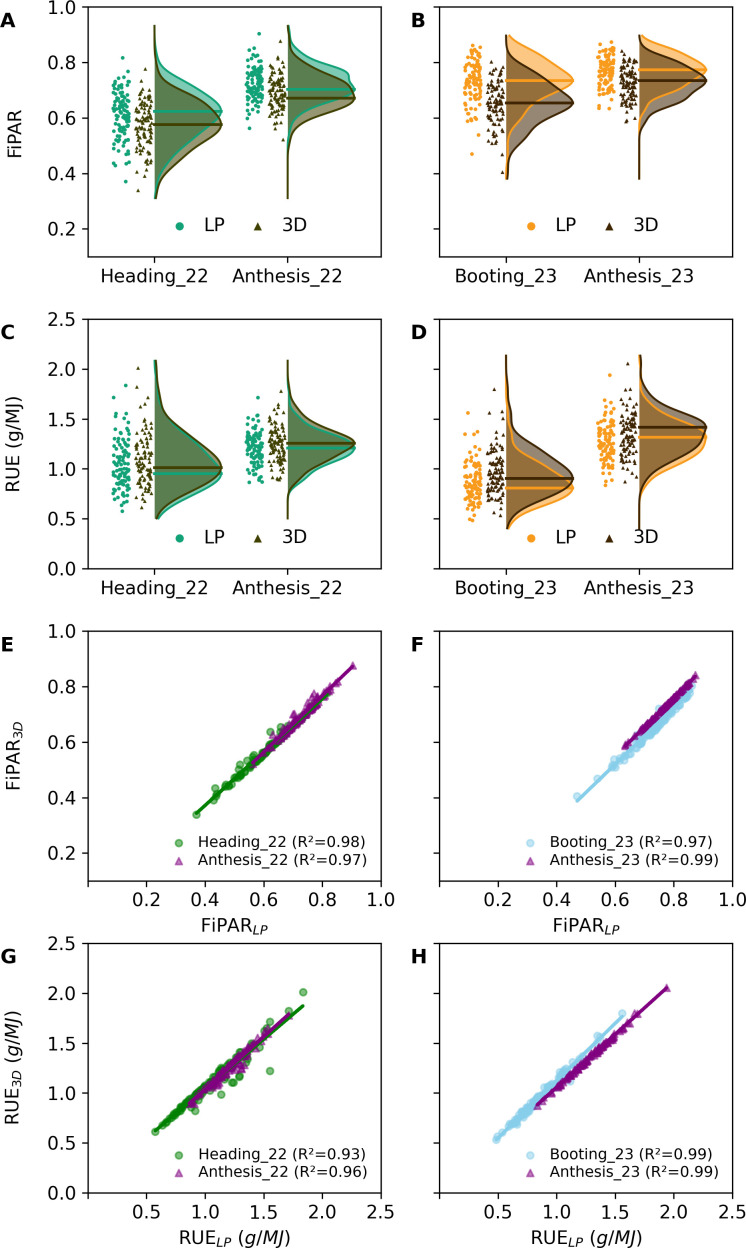
Distribution and comparison of FiPAR and RUE derived from 3D canopy models and LP80 (manual) measurements at heading and anthesis in 2022 **(A, C)**, and booting and anthesis) in 2023 **(B, D)**. **(E, F)** show the correlation coefficients between FiPAR_3D_ and FiPAR_LP_ while **(G, H)** are between RUE_3D_ and RUE_LP_ at two distinct crop growth stages in 2022 and 2023, respectively.

FiPAR_3D_ and RUE_3D_ were comparable to FiPAR_LP_ and RUE_LP_ across years and growth stages (**Figure 4**). FiPAR estimates showed strong correlations, ranging from 0.97 to 0.99 (**Figures 4E, F**). Despite these close associations, FiPAR_3D_ were consistently lower than those derived from FiPAR_LP_ across all growth stages and years (**Figures 4A, B**). On average, FiPAR_3D_ estimates were 5.66% and 8.79% lower in 2022 and 2023, respectively, compared to FiPAR_LP_ measurements across all growth stages. Similarly, RUE estimates were highly correlated (averaged R^2^ = 0.97), with R^2^ ranging from 0.93 to 0.99 (**Figures 4G, H**). RUE_3D_ estimates were consistently higher than those derived from RUE_LP_ across all growth stages and years by 4.11% and 8.95% in 2022 and 2023, respectively (**Figures 4C, D**).

Heritability estimates for FiPAR and RUE were medium to high under both LP- and 3D-based approaches (**Table 1**). Heritability was consistently higher at the booting and heading stages than at the anthesis stage in both years, with 3D-based estimates showing comparable or slightly higher than those derived from the LP approach. Specifically, both FiPAR_LP_ and FiPAR_3D_ showed a heritability of 0.77 at the heading stage and 0.61 at the anthesis stage in 2022. In 2023, FiPAR_LP_ showed 0.71 at the booting stage and 0.63 at the anthesis stage, with FiPAR_3D_ showing 0.64 at both stages. RUE heritability was similar between methods, with 0.75 and 0.69 for RUE_LP_, compared with 0.87 and 0.89 for RUE_3D_ at heading and anthesis stages in 2022. In 2023, RUE_LP_ was 0.81 at booting and 0.68 at anthesis, and the corresponding RUE_3D_ was 0.79 and 0.68. Overall, RUE_3D_ showed around 22.5% higher heritability estimates compared to RUE_LP_ in 2022, with no improvement observed on average in 2023.

**Table 1:** Heritability estimations for RUE and its related traits.

Year/trait	2022			2023		
	Heading	Anthesis	Physiological maturity	Booting	Anthesis	Physiological maturity
FiPAR_LP_	0.77	0.61	–	0.71	0.63	–
FiPAR_3D_	0.77	0.61	–	0.64	0.64	–
RUE_LP_	0.75	0.69	–	0.81	0.68	–
RUE_3D_	0.87	0.89	–	0.79	0.68	–
BIOMASS	0.79	0.79	–	0.86	0.69	–
HI	–	–	0.64	–	–	0.80

FiPAR, fraction of intercepted PAR; LP, LP80-based measurement; RUE, radiation use efficiency; HI, harvest index.

### 3.2 3D canopy models reveal two architectural phenotypes

**Figure 5:**
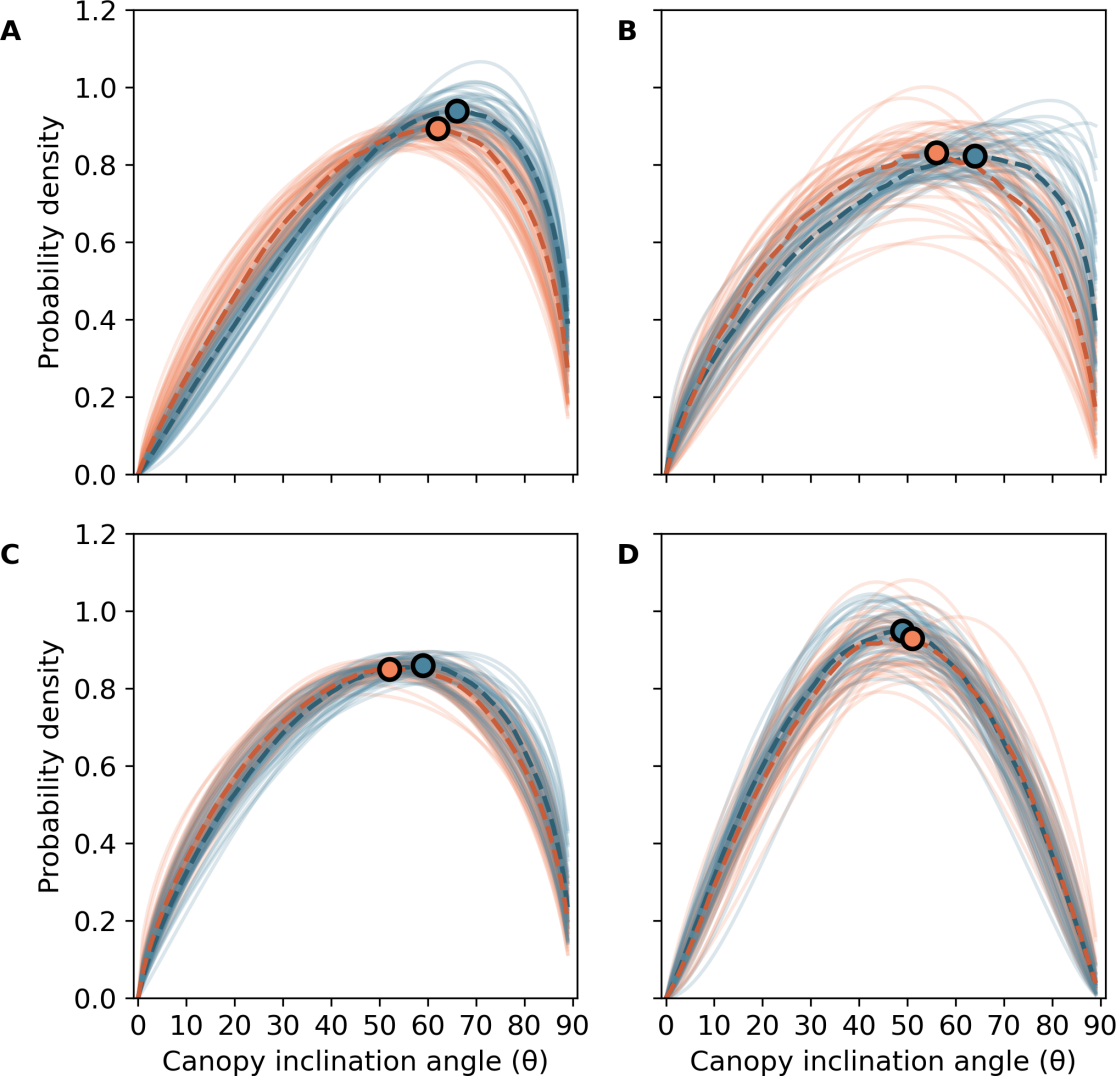
Fitted beta distributions of canopy inclination angles for all genotypes at the heading **(A)** and anthesis **(C)** stages in 2022 and at the booting **(B)** and anthesis **(D)** stages in 2023. Each solid-colored line represents one individual genotype. Blue lines represent erectophile phenotypes, while red lines indicate planophile phenotypes. Dotted lines show median values, and filled circles mark the maximum angles from the median values for each group.

Two distinct canopy architectural phenotypes were identified based on the distribution of canopy inclination angles across genotypes. One phenotype showed greater inclinations toward the right side (hereafter referred to as erectophile phenotypes), whereas the other showed lower inclination angles (hereafter referred to as planophile phenotypes). The distribution of canopy inclination angles shows temporal variations across growth stages in both years (**Figure 5**). Notably, the variation among all genotypes was lower at the anthesis stage than at the pre-anthesis stages. At the pre-anthesis stages (heading stage in 2022; and booting stage in 2023; **Figures 5A, B**), the density distribution of the canopy inclination angles followed a similar pattern, inclined towards the right-hand side, albeit with some subtle differences in the density peak and distribution. The erectophile phenotypes showed higher inclination angles than those of the planophile phenotypes across the canopy inclination angle range of 0° - 90°. With heterogeneous density distribution patterns at pre-anthesis stages across both years, the maximum median values were observed at 66° and 62°for the respective erectophile and planophile phenotypes at the heading stage in 2022. Similar density peaks at 64° and 56°for respective erectophile and planophile phenotypes were observed at the booting stage in 2023. However, density distributions became more homogenous for both architectural phenotypes at the anthesis stage (**Figures 5C, D**).

**Figure 6:**
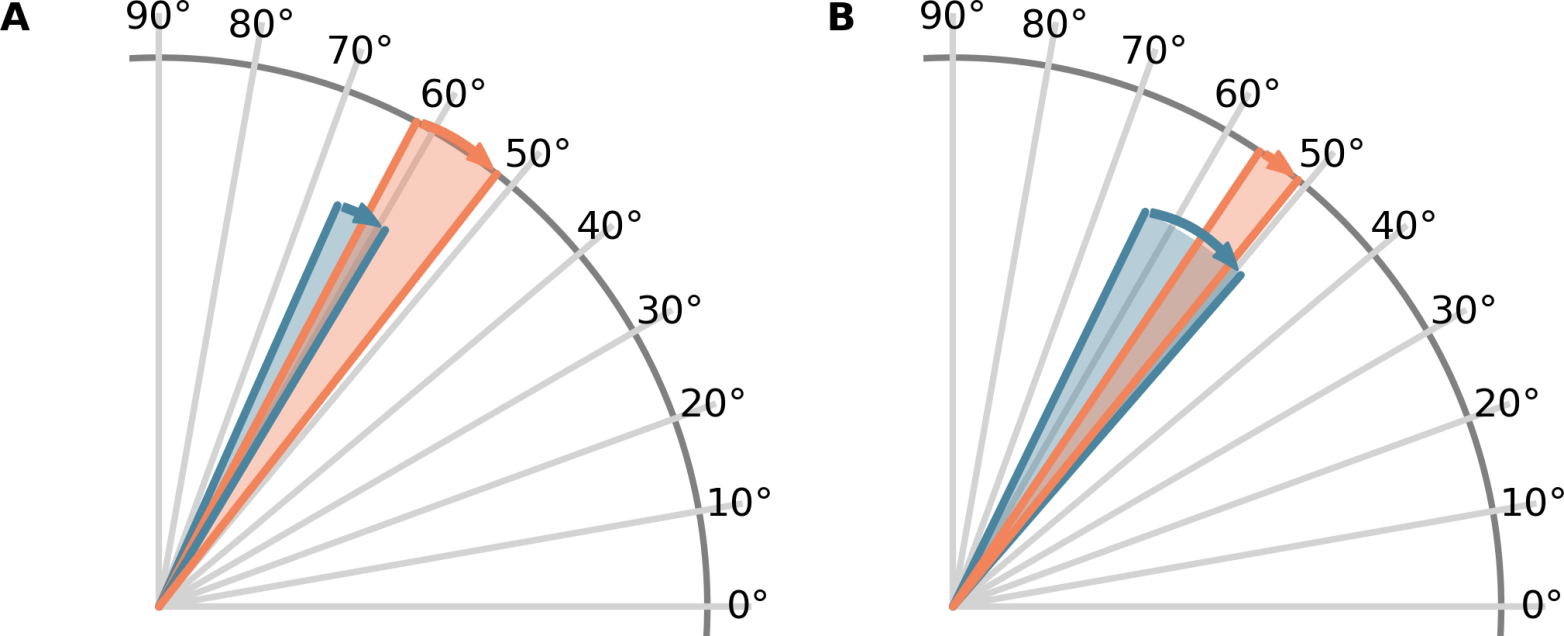
Changes in the median canopy inclination angles for two PA phenotypes between two growth stages. Blue lines indicate erectophiles, and red lines indicate planophiles in 2022 **(A)** and 2023 **(B)**. The arrow direction represents the phenological transition from the pre-anthesis to the anthesis stages.

Median canopy inclination angles decreased from pre-anthesis to anthesis stages: from 66° to 59° and from 62° to 52°for the erectophile and planophile phenotypes, respectively, in 2022 (**Figure 6**). Similarly, in 2023, median angles declined from 64° to 49° and from 56° to 51°for the erectophile and planophile phenotypes, respectively, between the booting and anthesis stages. Overall, median canopy inclination angles decreased from the pre-anthesis stage to anthesis in both phenotypes and both years.

### 3.3 Both erectophile and planophile phenotypes exhibit different trait responses

**Figure 7:**
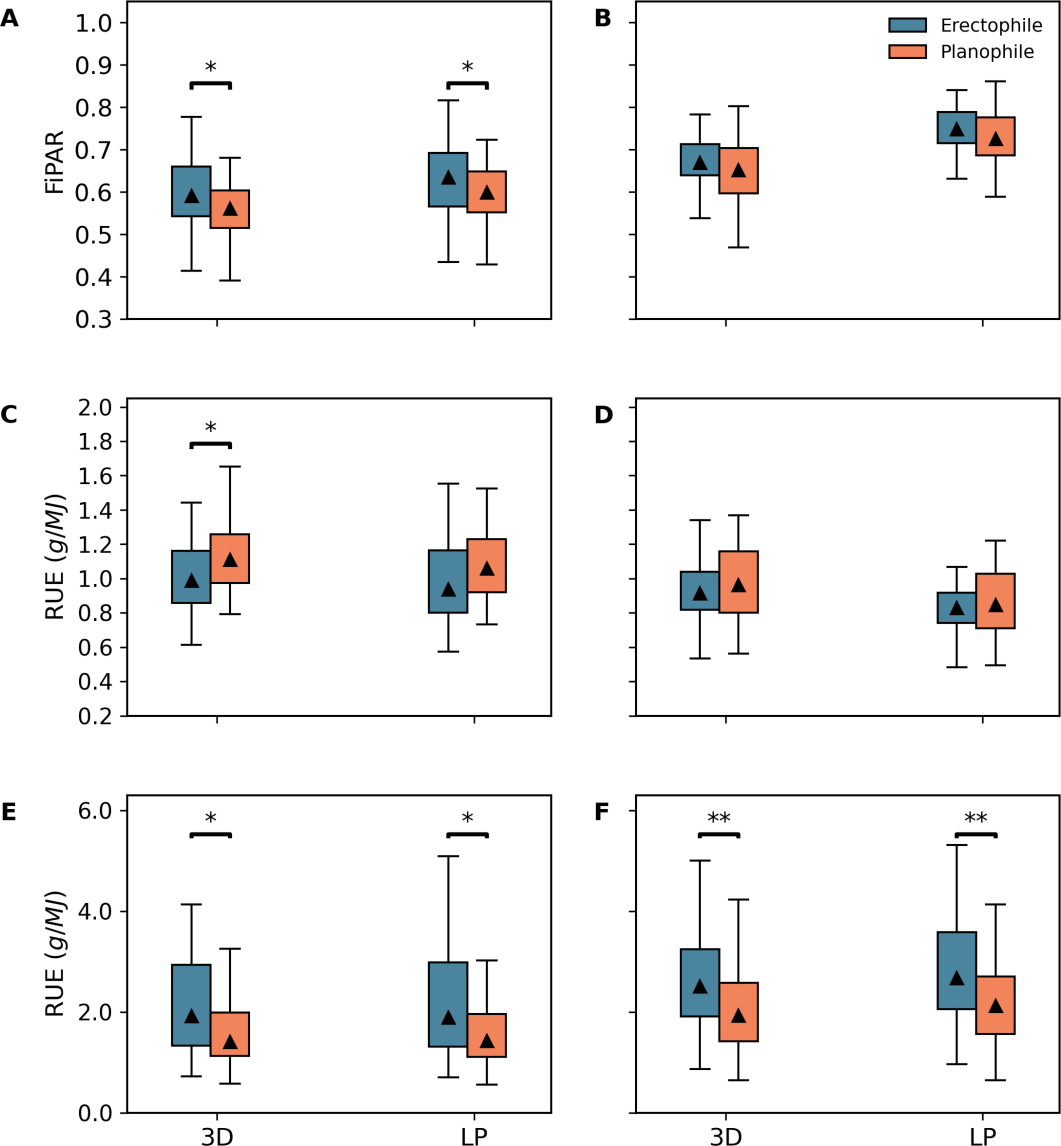
The FiPAR and RUE responses between two architectural phenotypes under 3D and LP80 methods. FiPAR **(A, B)** and RUE **(C, D)** were estimated at the heading in 2022 **(A, C)** and booting stage in 2023 **(B, D)**, respectively. Additionally, RUE is calculated for the period between the heading and anthesis stage in 2022 **(E)** and between the booting and anthesis stage in 2023 **(F)**. ▴ and * refer to the median values and statistically significant differences between the two PA phenotypes, respectively. (*p < 0.05, **p < 0.01).

Erectophile phenotypes showed higher FiPAR estimates than planophile phenotypes at the pre-anthesis stages (**Figure 7**), although this difference was statistically significance only in 2022 (*p* < 0.05, **Figure 7A**). On average, FiPAR estimates were 5.18% and 2.91% higher for erectophile phenotypes than for planophile phenotypes in 2022 and 2023, respectively.

RUE estimates showed different patterns between two architectural phenotypes at the pre-anthesis and after anthesis. During the pre-anthesis stages in both years, planophile phenotypes showed higher RUE estimates than erectophile phenotypes, although statistical significance was observed only for RUE_3D_ in 2022 (*p* < 0.05, **Figure 7C**). In contrast, RUE estimates under both methods showed an opposite pattern between the two PA phenotypes for the period between heading and anthesis stages in 2022 and booting and anthesis stages in 2023 (**Figures 7E, F**). Erectophile phenotypes exhibited a significantly higher RUE than planophile phenotypes (*p* < 0.05 in 2022, *p* < 0.01 in 2023), with increases of 36.17% and 32.17% in 2022, and 30.05% and 26.42% in 2023 for RUE_3D_ and RUE_LP_, respectively. These results suggest that plant architectural changes, such as canopy inclination angles, can significantly influence light interception and RUE during the crop growing period.

**Figure 8:**
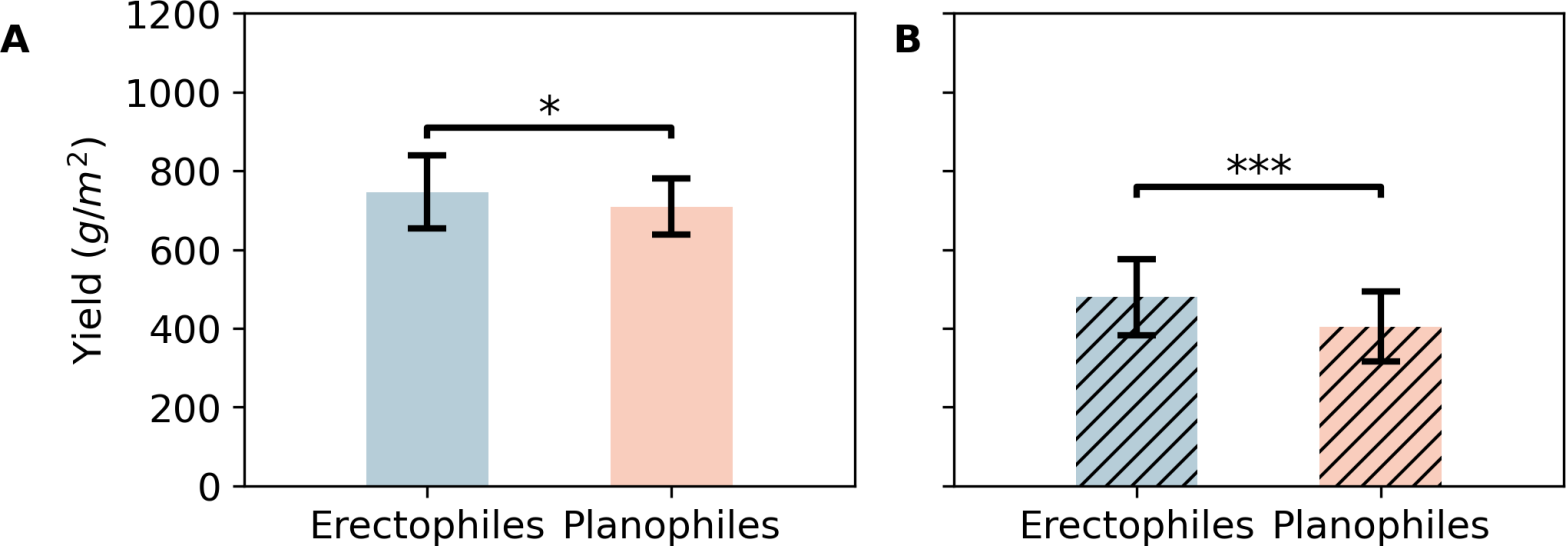
Comparative evaluation of grain yield between two architectural phenotype (erectophile and planophile phenotypes) in 2022 **(A)** and 2023 **(B)**. (*p < 0.05, ***p < 0.001).

Further, erectophile phenotypes showed significantly higher grain yield (5.2% and 18.6% in 2022 (*p* < 0.05) and 2023 (*p* < 0.001), respectively) than planophile phenotypes (**Figure 8**). Across the two years, erectophile phenotypes have a yield advantage (average across two years: 12%) over planophile phenotypes. Associated with these architectural differences, erectophile phenotypes were also 1.7% and 2.7% shorter plant height than planophile phenotypes in 2022 and 2023, respectively (*p* < 0.05; **Supplementary Figure 5**).

### 3.4 Traits derived from 3D canopy models provide additional information for grain yield prediction

**Figure 9:**
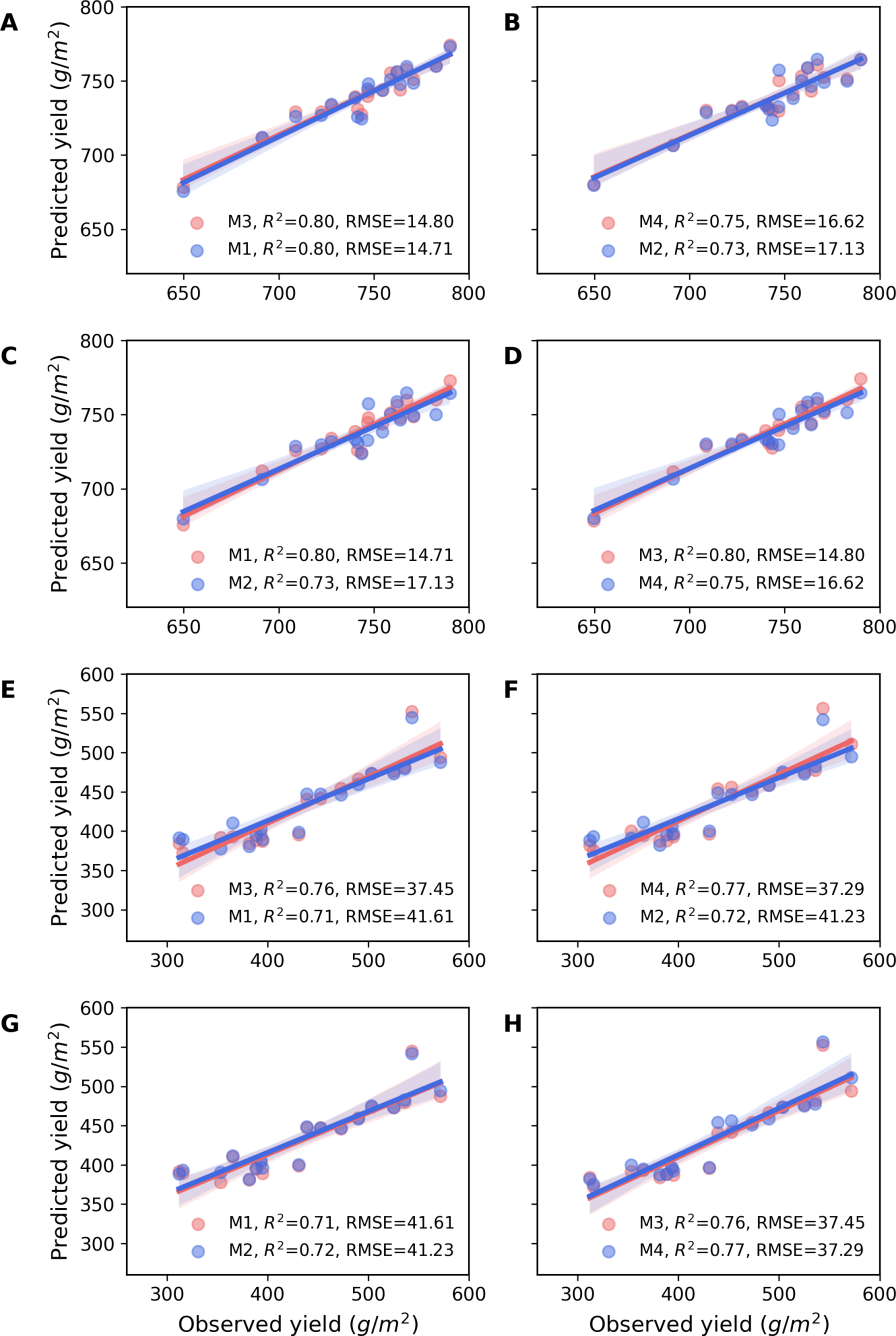
Correlation coefficient between the observed and predicted yield in 2022 **(A-D)** and 2023 **(E-H)**, respectively.

RF models using 3D-derived traits showed modest improvements in grain yield prediction compared with models using LP80-derived traits (**Table 2**; **Figure 9**). In 2022, the 3D model without PA (M1) achieved an RMSE of 2% lower than that of the LP80 model without PA (M2), with R^2^ increasing from 52% to 54%. When PA was included, the 3D model (M3) showed a further 1.6% reduction in RMSE relative to LP80 (M4), with R^2^ increasing from 53% to 55%. In 2023, the 3D model (M1) showed a slight RMSE reduction of 0.4% and a 1% increase in R^2^ compared with LP80 model (M2), whereas LP80 (M4) performed marginally better when PA was included, with RMSE reduced by 0.4% and R^2^ increased from 52% to 53%. In addition, inclusion of PA improved model performance under both methodological approaches in both years. For 3D-derived traits (M1 to M3), RMSE decreased by 1% in 2022 and by 2.1% in 2023, while R^2^ increased from 54% to 55% in 2022 and from 51% to 52% in 2023, respectively. For LP80-derived traits (M2 to M4), RMSE decreased by 1.4% in 2022 and by 2.9% in 2023, with R^2^ increasing from 52% to 53% in 2022 and from 50% to 53% in 2023.

**Table 2:** Comparison of model performances for four yield prediction models, averaged over 30 runs. Values are presented as mean ± standard deviation.

Metric	2022				2023			
	M1	M2	M3	M4	M1	M2	M3	M4
RMSE (g/m^2^)	20.7 ± 3.28	21.13 ± 3.15	20.5 ± 3.35	20.84 ± 3.28	50.05 ± 8.43	50.26 ± 8.35	48.98 ± 7.82	48.80 ± 7.76
R^2^ (%)	54 ± 14	52 ± 16	55 ± 14	53 ± 16	51 ± 11	50 ± 11	52 ± 13	53 ± 12

Although improvements in R^2^ were modest, the probability of superiority indicated that 3D-derived traits and PA reduced prediction error (**Supplementary Figure 8**), based on Equations 4–6. In 2022, 3D-derived traits reduced prediction errors more than LP80-based models, with probabilities of 0.55 without PA and 0.54 with PA. Inclusion of PA improved LP-based model performance consistently across years (0.55 in 2022; 0.54 in 2023). Even after accounting for PA, 3D-derived traits continued to reduce prediction error (0.54 in 2022; 0.53 in 2023), indicating that they may have provided additional fine-scale structural information beyond PA. In contrast, probabilities close to 0.5 in 2023 indicated no consistent pattern between approaches, suggesting that the relative contribution of 3D-derived traits varied across environmental conditions.

Among all four models, the best-performing model results highlight two important findings. First, traits derived from the 3D canopy models showed a modest improvement in predictive performance compared to those using LP80-derived traits, with differences in R^2^ of up to 7% (**Figures 9C, D**, G-H). Second, the inclusion of PA phenotypes was associated with modest increases in model performance across both years, with R^2^ differences of up to 5% across both years compared to models without PA (**Figures 9A, B, E, F**).

## 4 Discussion

This study assessed the use of canopy-based 3D models for quantifying variation in *K* and related physiological traits across diverse wheat genotypes under field conditions. Three main findings are discussed: (1) 3D canopy models-derived estimates were comparable with the conventional LP80 approach, (2) genotype-specific architectural phenotypes and temporal changes in canopy architecture across growth stages, and (3) 3D-derived architectural traits better contributed to yield prediction models compared to LP80-derived predictors. Together, these findings demonstrate the potential of 3D canopy models as an efficient and scalable approach for capturing subtle variation in architectural and physiological traits in wheat phenotyping.

### 4.1 Spatial and temporal variations in *K*_3D_ and its comparability with the LP80-based approach

The 3D canopy models revealed considerable variation in *K_3D_* among wheat genotypes (**Figure 3**). Unlike the manual LP80 approach, which uses a constant value (Campbell, 1986; Pokovai and Fodor, 2019), *K_3D_* was derived from genotype-specific canopy inclination angle distribution from 3D canopy models. This indicates that the 3D-derived approach captured variation in canopy architecture, particularly differences in canopy inclination angle distribution. Such genotypic differences in *K* are important to consider, as they lead to variation in canopy light attenuation and consequently influence estimates of FiPAR and RUE.

The lower FiPAR_3D_ values suggest that the 3D approach was more sensitive to canopy inclination angle and architectural heterogeneity, rather than simply producing a lower estimate of light interception. FiPAR_3D_ was consistently lower than FiPAR_LP_, with average differences of 5.66% and 8.79%, across the two years. This difference likely reflects the different basis of the two methods. The LP80 approach provides manual, vertically averaged point measurements of light interception and relies on simplified assumptions of canopy structure (Ratjen and Kage, 2016), whereas FiPAR_3D_ is estimated using genotype-specific *K*_3D_. Despite these differences, FiPAR_3D_ and FiPAR_LP_ showed a strong positive correlation across all growth stages and years (**Figures 4E, F**). This indicates that FiPAR_3D_ captured similar overall patterns of light interception to the manual LP80 approach, while also providing architecture-based information linked to genotype-specific canopy structure. Thus, FiPAR_3D_ can be considered complementary to FiPAR_LP_.

A similar interpretation applies to RUE. The 3D-derived estimates may retain physiological significance. RUE_3D_ and RUE_LP_ also showed a strong positive correlation across all growth stages (**Figures 4G, H**). Since RUE_3D_ was calculated from FiPAR_3D_, it also incorporated variation associated with genotype-specific canopy architecture. Further, the heritability of RUE_3D_ is approximately 11% higher over two years than that of RUE_LP_, consistent with the observations reported by Zang et al. (2023). Higher heritability in 3D-derived estimates may capture a greater proportion of genotype-dependent variation, supporting their usefulness for phenotyping application in breeding programs. In this context, determining genotype-specific canopy inclination angles can provide additional insights into complex physiological traits such as RUE.

These findings show that 3D canopy phenotyping can serve as a complementary approach, rather than replacing conventional measurements, by providing additional structural information that is not directly captured by the LP80-based method. In particular, the ability of 3D canopy models to quantify canopy heterogeneity, including canopy inclination angles and canopy structure may help improve the interpretation of physiological traits.

In addition to methodological differences between the two approaches, canopy inclination angle also showed temporal variation across developmental stages in this study. Interestingly, the average canopy inclination angle decreased as developmental stages progressed. In 2022, it was 65° at the heading stage and 54° at the anthesis stage, while in 2023, it decreased from 58° at the booting stage to 48° at the anthesis stage (**Figure 5**). These patterns imply that genotypes exhibit substantial plasticity in leaf angle, orientation, and distribution across crop developmental stages, likely to optimize resource-use efficiency (Reynolds et al., 2012). Since the changes in canopy inclination angle largely occurred during the pre-anthesis stages, it is reasonable to propose that the genetic architecture, or putative genes, controlling leaf orientation and angles may be developmental-stage- and environment-sensitive. However, such growth stage-specific genetic loci or genes need to be identified in cereals. Previous studies have shown that higher leaf inclination angles were correlated with the overexpression of genes involved in brassinosteroid and cytokinin biosynthesis in rice (Feng et al., 2016; Huang et al., 2023; Kim et al., 2023; Mantilla-Perez and Salas Fernandez, 2017). It would be interesting to investigate the temporal pattern of these genes’ expression and correlate with leaf angle changes during the pre-anthesis stages. Alternatively, an association genetics analysis could identify candidate genetic variants and their associated genes regulating leaf angle at or across different developmental stages, thereby identifying putative genes.

### 4.2 Architectural phenotypes derived from 3D canopy model show variable trait responses

Using 3D canopy models, two architectural phenotypes were identified: erectophile and planophile phenotypes. These two canopy architectures highlight the importance of architectural variation among genotypes in modifying canopy light interception and associated responses including FiPAR, RUE, and grain yield. This suggests that 3D-derived architectural phenotypes provide biologically meaningful information beyond simple canopy-level trait measurements.

The transition from erectophile phenotypes at pre-anthesis stages to more planophile phenotypes at anthesis indicates that canopy architecture was not static but changed during crop development. Consistently, this geometric alteration in leaf angles was also reported at the canopy level across different growth stages in wheat (Richards et al., 2019). Those studies showed the prevalence of erect canopy architecture during the pre-anthesis stages, whereas the predominance of floppy architecture (planophile phenotype) occurs at later growth stages. Such structural plasticity may help wheat genotypes adjust canopy structure across developmental and environmental conditions, thereby modifying vertical light distribution and improving resource-use efficiency.

These architectural differences were reflected in canopy physiological performance and yield-related responses. In this study, erectophile phenotypes showed a higher RUE and yield compared to planophile phenotypes. Such responses are likely linked to differences in canopy light distribution associated with canopy architecture. Erectophile phenotypes allow greater penetration of radiation into lower canopy layers while reducing light saturation in upper leaves, thereby improving whole canopy photosynthesis. This interpretation is supported by the significantly higher FiPAR observed in erectophile phenotypes at the heading stage in 2022 (**Figure 7A**) as well as their higher average LAI and intercepted PAR compared with planophile phenotypes., while planophile phenotypes showed slightly but not significantly higher intercepted PAR at anthesis across both years. (**Supplementary Figure 6**; **Supplementary Figure 7**). These observations are consistent with previous studies that reported erectophile phenotypes showing a higher RUE (24% (Long et al., 2006)), canopy dry biomass (11% (Zhi et al., 2022)) and grain yield (24% (Richards et al., 2019; Zhi et al., 2022)). Therefore, erectophile genotypes may not intercept a greater total PAR over the entire crop cycle, but their leaf orientation facilitates more uniform light distribution during the pre-anthesis stage within the canopy vertical profile, contributing to the accumulation of stem and leaf reserves. Together, these findings highlight that 3D-derived architectural phenotypes capture meaningful variation in canopy physiological performance and yield. The ability of 3D canopy models to quantify these architectural phenotypes and their temporal dynamics provides a useful framework for detecting structural variation that is closely linked to physiological traits and yield performance. Nevertheless, the contrasting responses observed between architectural phenotypes may also partly reflect differences in seasonal weather conditions and the genotype sets evaluated in each year. Future studies using common genotypes across environments would help clarify genotype × environment effects on canopy architecture and RUE estimation.

The relative advantage of erectophile and planophile phenotypes also depends on the environmental conditions. Under favorable environments, erectophile phenotypes have substantial potential to enhance crop photosynthesis and increase grain yields through greater crop biomass, slower rate of canopy senescence, and higher grain yields (Richards et al., 2019). In contrast, under less favorable environments (such as low-light and cloudy conditions), planophile phenotypes may be more effective and advantageous, intercepting more light and achieving higher RUE, particularly when LAI has not yet reached its saturation point before anthesis. This pattern was also observed in this study, where planophile phenotypes showed higher RUE than erectophile phenotypes under lower incident PAR in both years (**Figure 1**, **Figure 7**). In the early stages of crop growth, planophile phenotypes can achieve rapid canopy closure by capturing more light from the top layers despite lower FiPAR across the entire canopy, contributing to the accumulation of carbohydrates in the stem and leaf sheath. These stored carbohydrates are then remobilized to support grain filling during the post-anthesis stage. Using CN-Wheat models, it has been predicted that the planophile phenotypes during the pre-anthesis stages accumulate more carbon (Barillot et al., 2019), potentially due to a higher RUE. Further, cooler environments during the early growth stages may slow leaf growth and lower LAI, under which the planophile phenotype could provide extended leaf surface area and achieve a higher canopy photosynthesis than the erectophile phenotype. Therefore, planophile phenotypes may provide a physiological advantage during early growth stages, particularly under cooler and low-radiation environments.

### 4.3 Traits derived from 3D canopy models improve grain yield predictions

To evaluate the contribution of architectural trait information to yield prediction, RF prediction models were used to compare the roles of 3D-derived traits and PA. Overall, models based on 3D-derived traits performed within a similar range to LP80-based models, with only modest differences in RMSE and R^2^ across years. This suggests that 3D-derived traits incorporate spatial canopy heterogeneity, which may explain subtle differences even when overall prediction performance remains comparable. Similar contributions of canopy architectural traits to yield prediction have been reported previously (Miao et al., 2021; Paulus, 2019).

Incorporating PA into the models led to additional changes in performance under both approaches. This indicates that architectural classification provided additional information beyond FiPAR and RUE alone. The relatively larger effect of PA in some scenarios, particularly in 2023, suggests that architectural classification may be particularly informative under heterogeneous environmental conditions where genotype × environment interactions influence canopy structure.

Overall, the magnitude of performance differences across models remained modest, but 3D-derived traits provided complementary structural information. The probability of superiority further suggests that 3D traits and PA reduced prediction error for different subsets of genotypes. Therefore, the main value of 3D canopy models lies not simply in improving overall prediction accuracy, but in providing architecture-based traits that help explain genotype-specific variation in yield performance. Although 3D phenotyping requires additional processing, its strength lies in providing quantitative and repeatable measurements of subtle canopy characteristics that are difficult to assess manually in breeding programs.

## 5 Conclusion

This study demonstrated that 3D canopy models derived from a mobile robotic phenotyping system can quantify canopy *K*, architectural phenotypes, and related physiological traits in field-grown spring wheat. The approach captured genotype-specific and temporal variation in canopy architecture, including erectophile and planophile phenotypes, which helped explain differences in RUE and grain yield. Although 3D-derived traits showed comparable performance to LP80-based traits, their value lies in providing additional structural information that is difficult to obtain from manual measurements alone. Overall, 3D canopy phenotyping offers a complementary tool for characterizing canopy structure and associated traits under field conditions, while reducing manual labor requirements through robotic platform and providing high-resolution, quantitative datasets with improved measurement consistency.

## Data Availability

The raw data supporting the conclusions of this article will be made available by the authors, without undue reservation.
