# Supplementary material for: Field-scale robotic phenotyping of three-dimensional wheat canopy architectural traits
Source: Front Plant Sci. 2026 Jul 1;17:1856006. doi: 10.3389/fpls.2026.1856006 (PMC13368997; doi:10.3389/fpls.2026.1856006)
Supplement: Supplementary file 1 [file DataSheet1.docx]

**Supplementary materials**

**Two-parameters beta distribution function**

|  | $f(t)=\frac{1}{B(\mu,\nu)}\left( 1-t \right)^{\mu-1}-t^{\nu-1}$ | (S1) |
| --- | --- | --- |

Where represents the ratio of $2\theta_{L}$ to π. $f(t)$is the leaf inclination probability density. The beta distribution $B(\mu,\nu)$ is defined as:

|  | $B(\mu,\nu)=\int_{0}^{1} \left( 1 - x \right)^{\mu-1}x^{\nu-1}dx=\frac{\Gamma(\mu)\Gamma(\nu)}{\Gamma(\mu+\nu)}$ | (S2) |
| --- | --- | --- |

Where $\Gamma$is the gamma function and two parameters $\left( \mu, \nu\right)$are used for the beta distribution as:

|  | $\mu=(1-\bar{t})\left( \frac{\delta_{o}^{2}}{\delta_{t}^{2}}-1 \right)\text{ }\nu=\bar{t}\left( \frac{\delta_{o}^{2}}{\delta_{t}^{2}}-1 \right)$ | (S3) |
| --- | --- | --- |

Where $\bar{t}$ is an expected mean and $\delta_{t}^{2}$is the variance of$t$. $\delta_{o}^{2}$represents the maximum standard deviation.

**
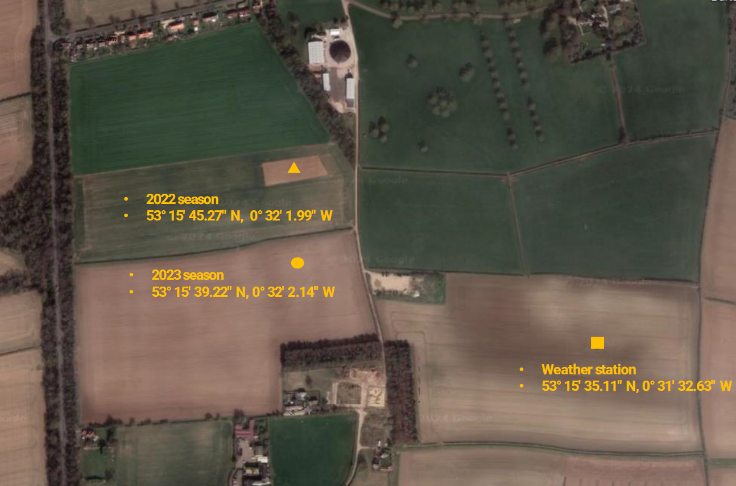
**

Supplementary Figure 1. Two field trial sites in 2022 (represented by a triangle (▲) and 2023 (●) in Google Earth. The weather station is indicated by a square (■).

**
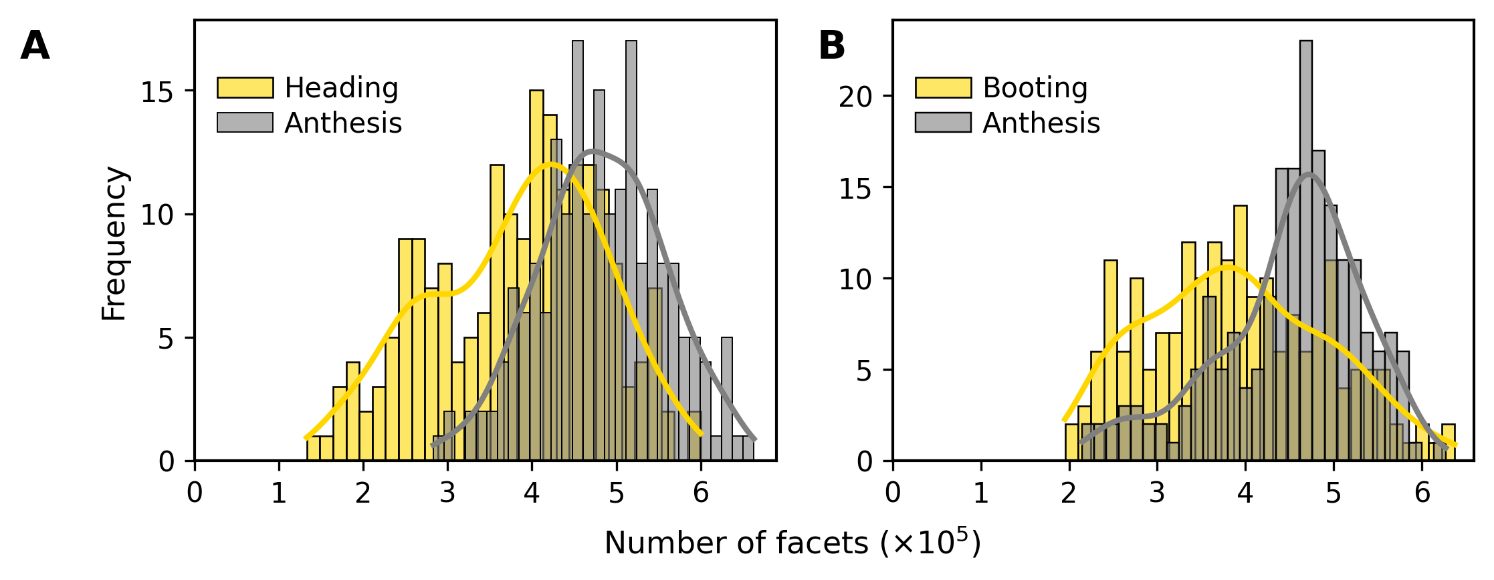
**

Supplementary Figure 2. The distribution of the number of facets for all genotypes in 2022 (A) and 2023 (B). Yellow and grey colors indicate the heading and anthesis stages in (A), and the booting and anthesis stages in (B).


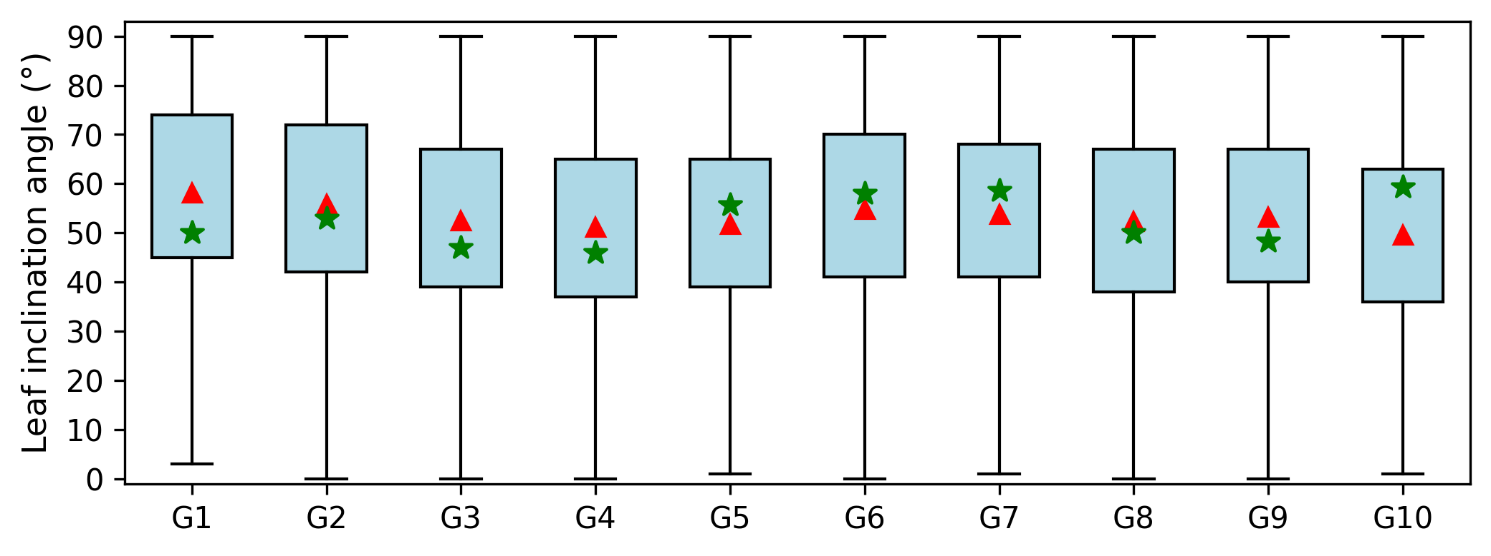


Supplementary Figure 3. Validation of facet angle (canopy inclination angle) across ten genotypes (G1-G10) in a mini-pot experiment. The boxplot displays the variation in 3D-measured canopy inclination angles. The red triangle represents the median canopy inclination angle from 3D data, while the green star indicates the median angle measured manually using a protractor.


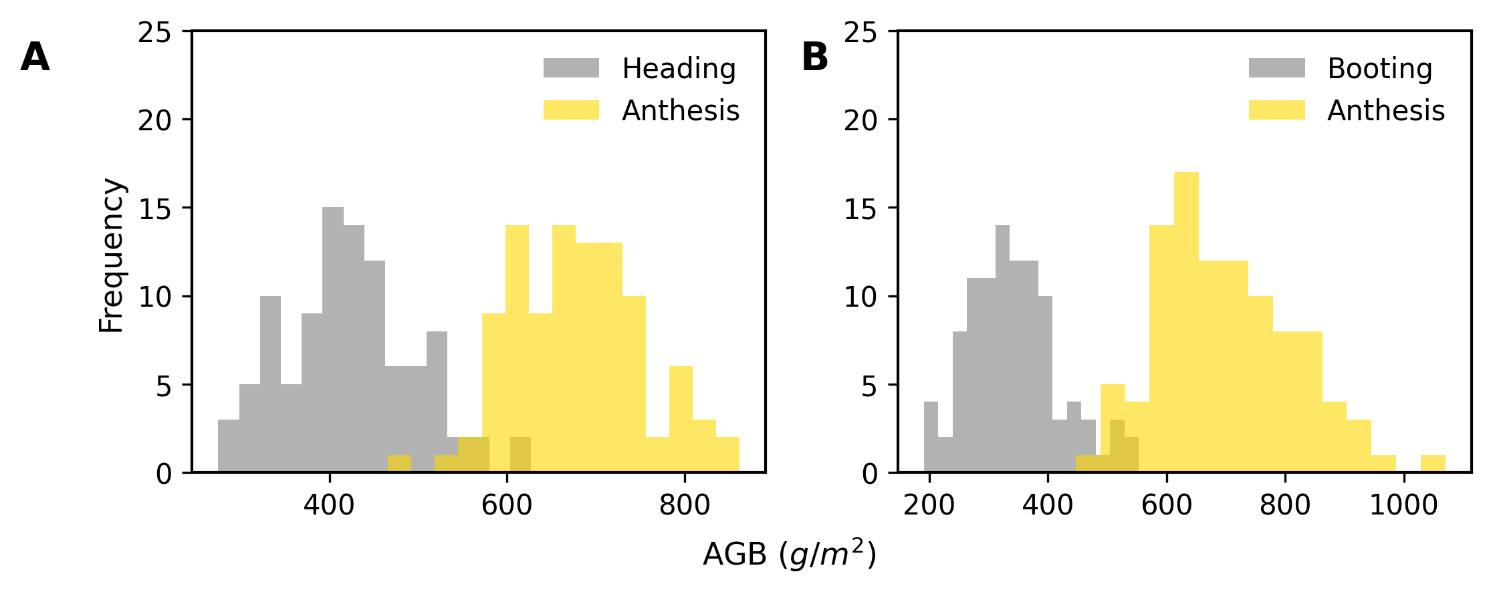


Supplementary Figure 4. The distribution of the dried biomass across all genotypes in 2022 (A) and 2023 (B).


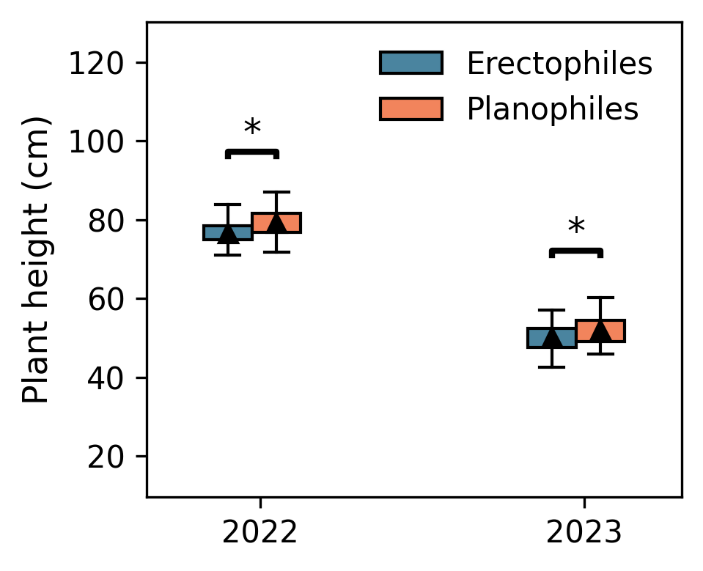


Supplementary Figure 5. A comparative evaluation of plant height at the physiological maturity stage between two PA phenotypes (erectophile vs planophile phenotypes). ▲represents the medium value of each phenotype. (* *p* < 0.05).


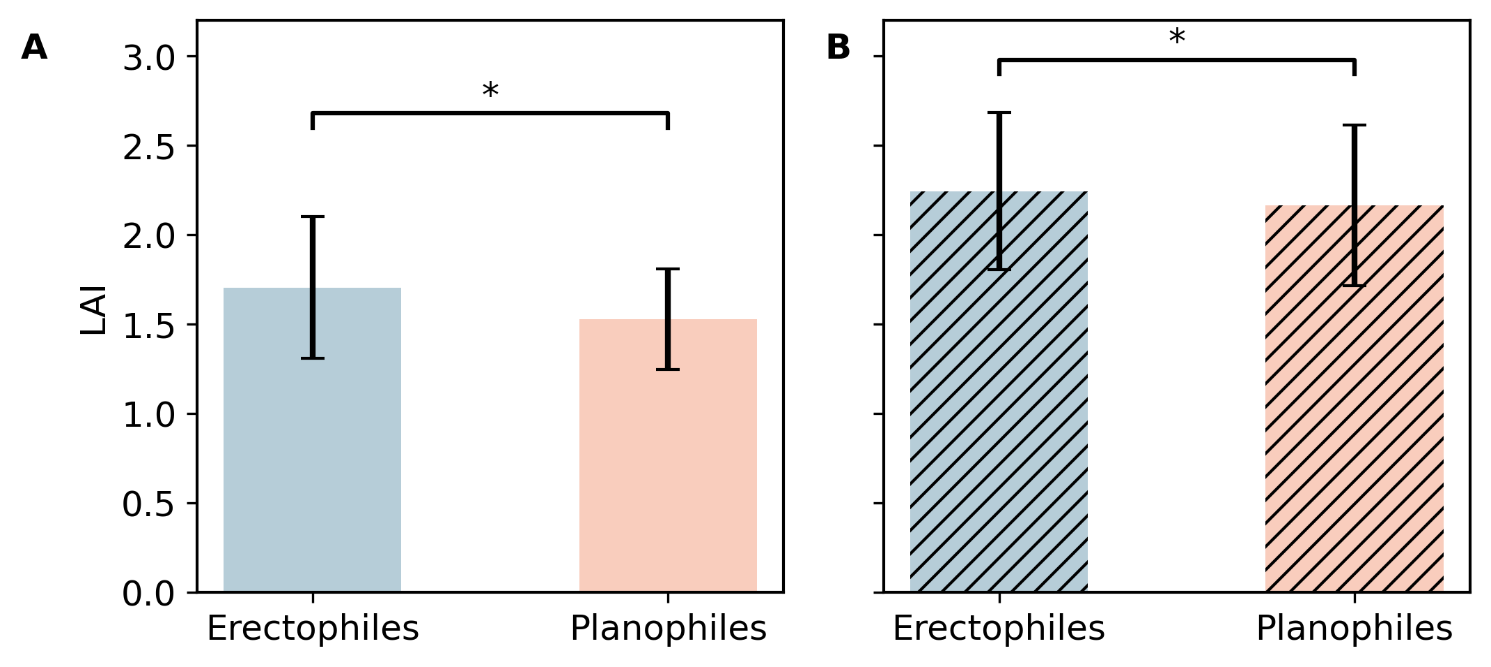


Supplementary Figure 6. Comparison of LAI between erectophile (blue) and planophile (red) phenotypes in 2022 (A) and 2023 (B). Error bar represents standard deviation. (* *p* < 0.05).


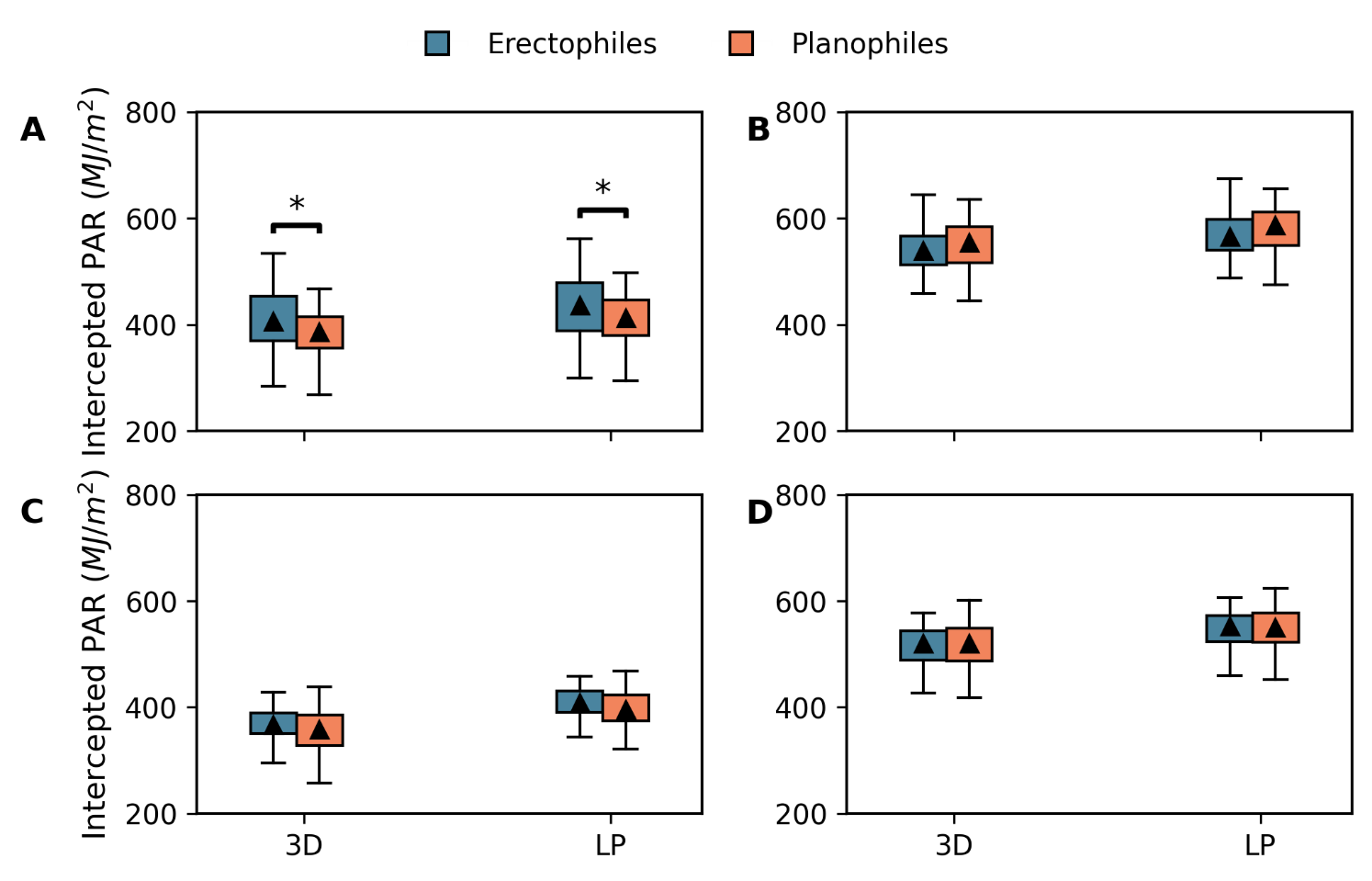


Supplementary Figure 7. Comparison of intercepted PAR between erectophile (blue) and planophile (red) phenotypes in 2022 (A: heading; B: anthesis stage) and 2023 (C: booting; D: anthesis stage). (* *p* < 0.05).

Predictive performance between modelling approaches was compared using the probability of superiority, defined as the proportion of samples for which a candidate model produced lower absolute prediction error than a reference model. For each sample, absolute prediction error was calculated as

|  | $\left\vert error\vert\text{ }=\text{ }\vert y_{obs}-y_{pred} \right\vert$ | (S4) |
| --- | --- | --- |

Where y_obs_ is the observed value and y_pred_ is the predicted value.

A binary improvement indicator was then defined for each paired observation:

|  | $I=\text{ }\left\{ \begin{matrix} 1, & \left\vert error_{candidate}\vert\text{ }<\text{ }\vert error_{baseline} \right\vert\\ 0, & otherwise \end{matrix} \right.$ | (S5) |
| --- | --- | --- |

Where *I* is the improvement indicator.

For each training run containing n samples, the probability of superiority was computed as:

|  | $P=\frac{1}{n}\sum_{i=1}^{n} I_{i}$ | (S6) |
| --- | --- | --- |

Where *P* is probability of superiority, *n* is the number of samples within a run.

*P* represents the proportion of cases in which the candidate model achieved lower absolute error than the baseline model. The resulting probabilities were calculated for each run and summarized across runs for each model comparison and year.


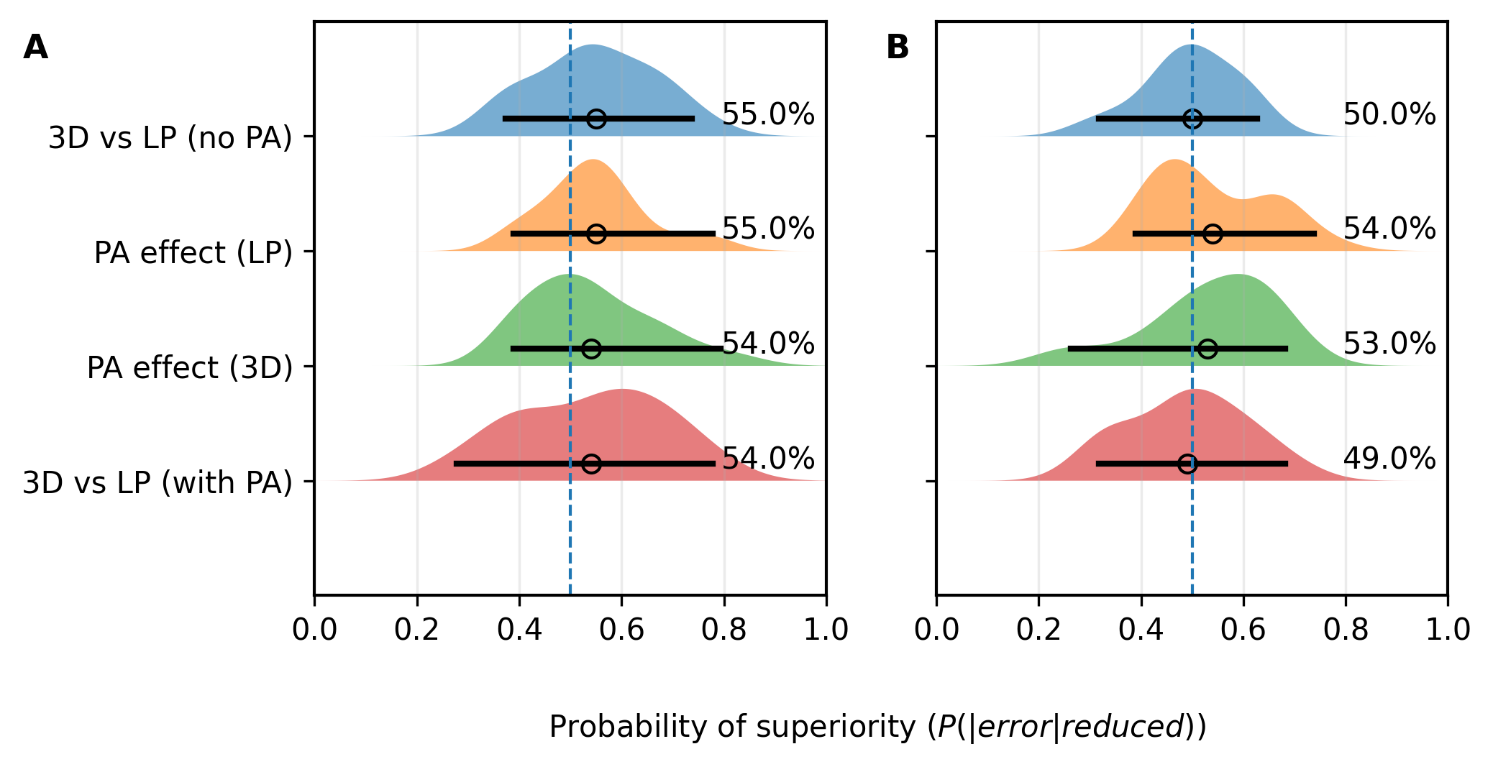


Supplementary Figure 8. Probability of superiority across model comparisons between 3D-derived and LP-derived models, with and without PA in 2022 (A) and 2023 (B). Open circles indicate the mean probability estimate. A blue vertical reference line denotes equal performance between models.
